# Notes and News

**Published:** 1901-05-04

**Authors:** 


### Notes and News.

A second International Congress of the Medical Press will be held at Brussels on September 20th.

The appearance of small-pox in various places between Liverpool and Leicester has been traced to a Mormon Conference held in Nottingham on March 24th.

The annual report of the Registrar-General for England and Wales shows continuous increase in the marriage rate, and continuous decrease in the birth rate. The decrease in the birth rate is conspicuous amongst the well-to-do. Amongst the poorest classes, where the births are most numerous, the infantile death rate is the highest, which adds to the present unsatisfactory aspect of population statistics.

The new Isolation Hospital at Rush Green, in Dagenham parish, which has been built for the Romford Urban and Rural District Councils, is now open. The buildings include two main blocks with ten beds in each ward, and two recovery wards with two beds in each. The cost has worked out at £300 per bed. Messrs. Coulson and Lofts of Cambridge built the hospital from designs of Mr. F. Whitmore, of Chelmsford.

A public meeting has been held in Belfast with the view of securing the better equipment of Queen's College. Lord Londonderry, Lord Dufferin, the Mayor of Belfast, and many other influential personages attended. The Right Hon. W. J. Pirrie and Sir James Musgrave, Bart., both of whom were present, have promised assistance. Mr. Pirrie offers a new laboratory of natural philosophy and engineering, and Sir James Musgrave promises £5,000. The Lord Mayor will give recreation grounds, Mr. J. McConnel £1,000, and the medical professors also promise £1,000. The meeting gave the superintendents of the various departments the opportunity to urge the claims of the particular branch of knowledge with which each was concerned, and the immediate result of the discussions was the establishment of a chair of pathology which will be occupied by Dr. Lorrain Smith.

As in former years, the present report of the Leicester Infirmary shows the working people of that city to be exceptionally generous in supporting their hospital.

The Parker Memorial Prize is a prize of 75 guineas awarded in open competition to the writer of an essay on a given subject who is a medical officer of the Army, Navy, or India services of executive rank on full pay. It is awarded every third year. Captain Howell, of the Royal Army Medical Corps, gained the prize in the last competition.

The Christian Scientist is not to have it all his own way. At this very moment a society is being formed for the study of psychic science in its relation more especially to the alleviation of suffering and cure of disease. The society is to be called The London Psycho-therapeutic Society. The title is attractive from its all-embracing character. It is to provide a "favourable opportunity for the systematic study of what is known as animal magnetism, mesmerism, hypnotism, psycho-magnetics, &c." In the etceteras we must, presumably, include the therapeuticisms which are not mentioned amongst the other isms. The society, according to the circular issued, will not be in any sense "antagonistic to existing scientific and sectarian bodies." Is this meant to propitiate the Royal College of Surgeons and Christian Science at the same time? The circular bears the names of Mrs. J. Stannard, of Upper Baker Street, and of Mr. Arthur Hallam, of Newington. No one else is mentioned.

Dr. William Collingridge, M.A., M.D., LL.M., the newly-appointed Medical Officer of Health of the City of London, is the eldest son of Mr. W. H. Collingridge, of Enfield and the *City Press*, Aldersgate Street. He was educated at St. Bartholomew's Hospital, and Christ's College, Cambridge, at which University he took the degrees of M.A., M.B., D.P.H., and M.D., and subsequently that of LL.M. During the Turco-Servian War, in 1876, Dr. Collingridge, then a student, went out to Belgrade as a volunteer surgeon, and received a commission as surgeon, and left for the army before Novi-Bazar. He there organised the hospitals. Dr. Collingridge entered upon this duty without pay, appointment, or companion. At the termination of the struggle Dr. Collingridge was decorated by King Milan with the order of the Takova. He was appointed to the post of Medical Officer of the Port of London, and in this capacity he has been engaged for the past twenty years in the inspection of vessels entering the river, the examination of suspected cases, the disinfection of vessels, and the taking of other precautions against the introduction of cholera, plague, and other infectious diseases. Dr. Collingridge was appointed the Milroy Lecturer to the Royal College of Physicians in 1897, Examiner in State Medicine to the University of Cambridge in 1898, and the official delegate of the University of Cambridge to the International Congress of Hygiene held in Paris last year. He is a Fellow of the Incorporated Society of Medical Officers of Health, a Fellow and Member of the Council of the Sanitary Institute, a Fellow of the Chemical Society, Chairman of the Church Sanitary Association, Honorary Physician to the Printers' Almshouses, &c. He is the author of "Occurrence and Significance of Hæmorrhage in Typhoid Fever," "Scurvy in the Mercantile Marine," "Water and Filtration," "Quarantine in England," "Duties of Sanitary Inspectors," and other works.

